# Arthroscopic Circumferential Lateral Meniscus Reconstruction Using Pull-Through Technique and Anterior Half Peroneal Longus Tendon Autograft

**DOI:** 10.1016/j.eats.2025.103964

**Published:** 2025-11-02

**Authors:** Junming Wan

**Affiliations:** Department of Sports Medicine, The Seventh Affiliated Hospital, Sun Yet-sun University, Shenzhen, Guangdong, China

## Abstract

Total meniscectomy for irreparable meniscal tears results in kinematics of the lower limb and early osteoarthritis of the knee. Some strategies for reconstruction of the meniscus include meniscus allograft transplantation and meniscal scaffold replacement. Although meniscus allograft transplantation is widely used, it has limitations such as the graft sources, high costs, and high risk of disease transmission. The research on meniscal scaffold replacement is still in the animal experimental stage, and there is a lack of large-scale clinical trial data. In this Technical Note, the lateral meniscus is reconstructed by using the pull-out technique with anterior half autologous peroneal longus tendon. This technique provides a simple and efficient method for the reconstruction of the total meniscus.

Total meniscectomy results in high risk of osteoarthritis and poor functional outcomes.^1^ The 2 main approaches to meniscal reconstruction are meniscus allograft transplantation (MAT) and meniscal scaffold replacement (MSR). MAT has been popular for many years. Several researchers have reported good clinical outcomes after MAT.^2^ The high costs and limited source of allograft have restricted the widespread use of MAT. MSR supports tissue ingrowth and degrades over time, eliminating the need for donor tissue, although the high costs and slow scaffold integration may limit its use. Autologous tendons have advantages, including that they are easy to obtain, have good biocompatibility, are low cost, and exhibit remodeling similar to the meniscus.^3^ In this Technical Note, a technique for arthroscopic circumferential reconstruction of the lateral meniscus by using pull-through technique with anterior half autologous peroneal longus tendon (APLT) (Video 1) is described. Tips and Pitfalls of This Technique of our technique are listed in Table 1.

**Table 1:** Tips and Pitfalls of This Technique

Tips
Ensure adequate width and length for proper tendon graft placement and fixation.
Precisely position the tibial tunnel for optimal graft placement.
Fix the posterior root first, adjust tension to restore hoop stress, and reinforce the capsule with appropriate sutures.
Pitfalls
Maintain sufficient distance between bone tunnels for correct placement.
Choose appropriate suturing techniques for circumferential fixation to avoid damaging nerves and veins.
Properly secure the APLT graft and adjust tension to prevent extrusion, ensuring long-term stability.

APLT, autologous peroneal longus tendon.

## Surgical Technique

### Arthroscopic Evaluation and Debridement

**Fig 1:**
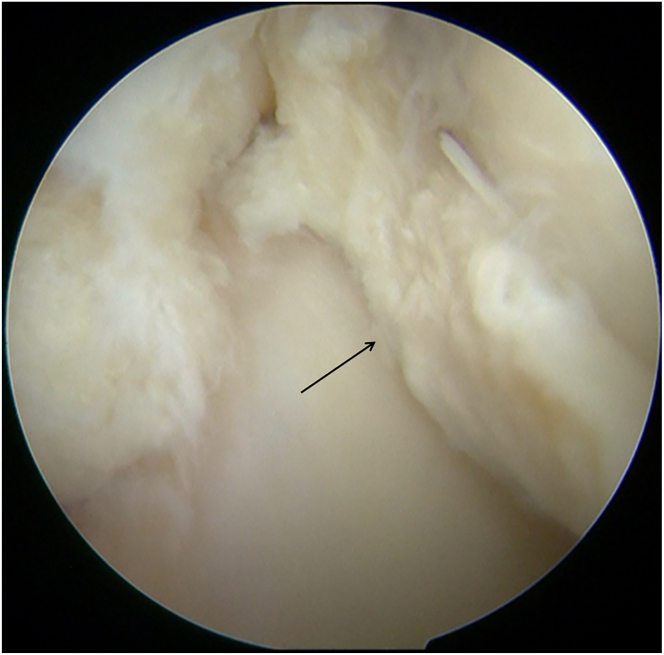
Arthroscopic screenshot of a left knee (anterolateral portal) shows a severe lateral meniscal tear.

**Fig 2:**
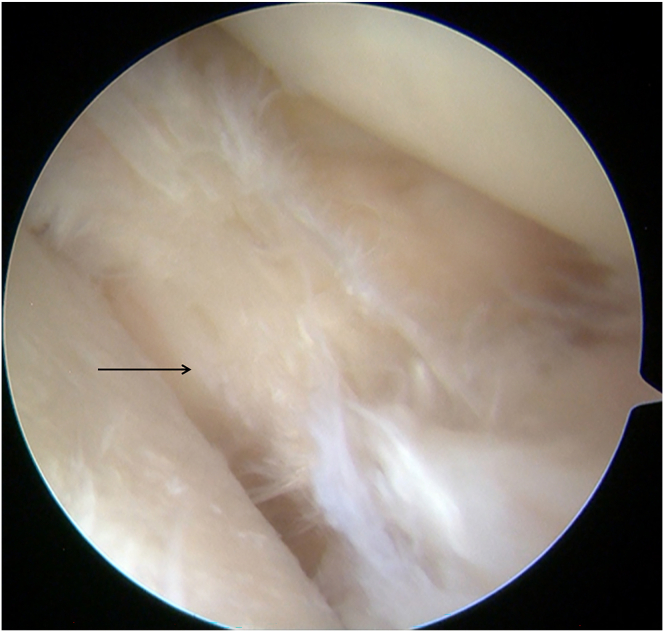
The remaining lateral meniscal tear margins (left knee, anterolateral portal) are debrided.

Diagnostic arthroscopy (Smith & Nephew) is performed through the anterolateral portal and anteromedial portal. With a probe (Smith & Nephew), the lateral compartment is carefully inspected, revealing a severe lateral meniscal tear with a fragment incarcerated in the intercondylar notch and deemed irreparable (Fig 1). The incarcerated portion is excised using a basket punch (Smith & Nephew). The remaining tear margins are debrided, the posterior root remnant is preserved, and the highly vascular tibial attachment bed is gently refreshed to optimize biological conditions for subsequent meniscal reconstruction (Fig 2).

### Preparation of the APLT

A longitudinal 2-cm incision is made on the posterior lateral ankle styloid process to expose the peroneus longus tendon. The anterior half of the tendon is harvested (6-mm diameter tendon stripper; Smith & Nephew), with the muscular tissue meticulously removed. The retrieved graft measures approximately 20 cm in length and 4 mm in diameter. The APLT is stitched with high-strength sutures (Rejoin) to facilitate later passage and fixation.

### Preparation of the Tibial Tunnels

**Fig 3:**
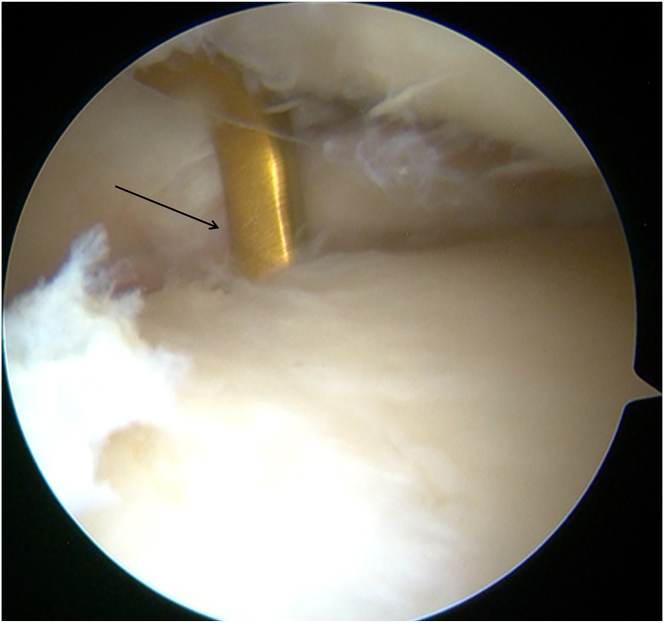
A tibial-tunnel ACL reconstruction guide (Rejoin) is positioned on the footprint of the posterior root (left knee, anteromedial portal).

**Fig 4:**
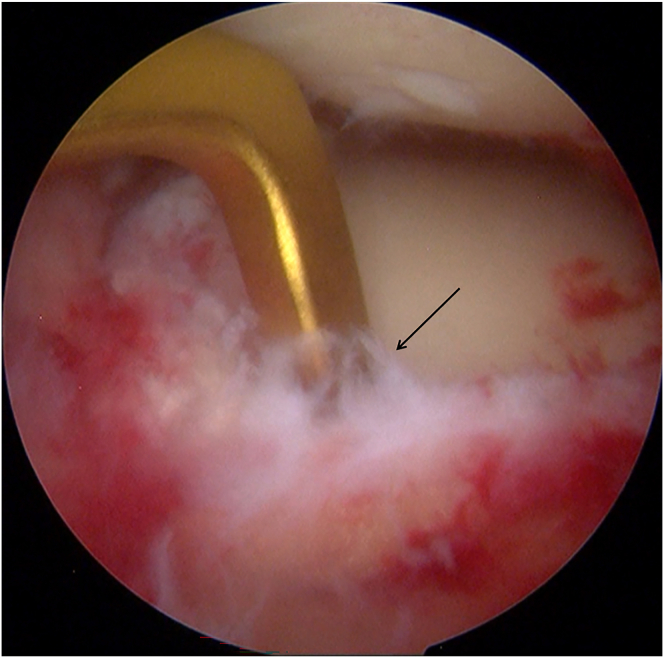
The tibial-tunnel ACL reconstruction guide (Rejoin) is repositioned at the footprint of the lateral meniscal anterior horn (left knee, anteromedial portal).

A 3-cm longitudinal skin incision is made medial to the tibial tuberosity. Through the anteromedial arthroscopic portal, the tibial footprints of both the anterior and posterior roots of the lateral meniscus are identified. A tibial-tunnel ACL reconstruction guide (Rejoin) is introduced via the medial incision and positioned on the footprint of the posterior root (Fig 3); a guide pin (Shuangyang) is advanced through the guide. After arthroscopic confirmation of the pin position, a 4.5-mm cannulated reamer(Rejoin) is used to create the posterior-root tibial tunnel. Using the same technique, the guide is repositioned at the footprint of the lateral meniscal anterior horn (Fig 4). A second guide pin is drilled, and a 4.5-mm cannulated drill is passed over the pin to ream the anterior-root tunnel, completing the dual-tunnel configuration.

### Graft Passage

**Fig 5:**
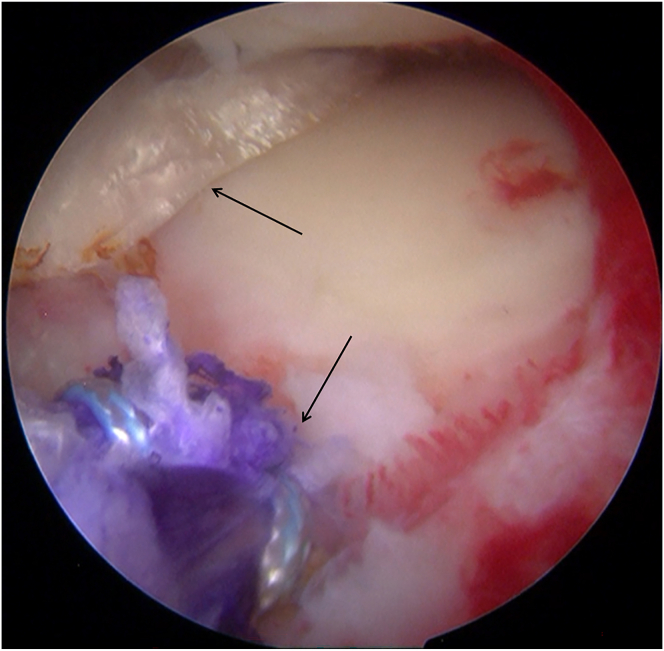
The posterior end of the graft is then fed through the retrieved loop, enabling the graft to be drawn into the tunnel using a shuttle-relay maneuver.

A passing pin loaded with a polydioxanone (PDS-II; Ethicon) loop is advanced through the posterior-root tibial tunnel, leaving the suture loop inside the joint. Via the anterolateral working portal, a grasper retrieves the loop and delivers it outside the joint. The PDS-II sutures attached to the posterior end of the graft are then fed through the retrieved loop, enabling the graft to be drawn into the tunnel using a shuttle-relay maneuver (Fig 5).

**Fig 6:**
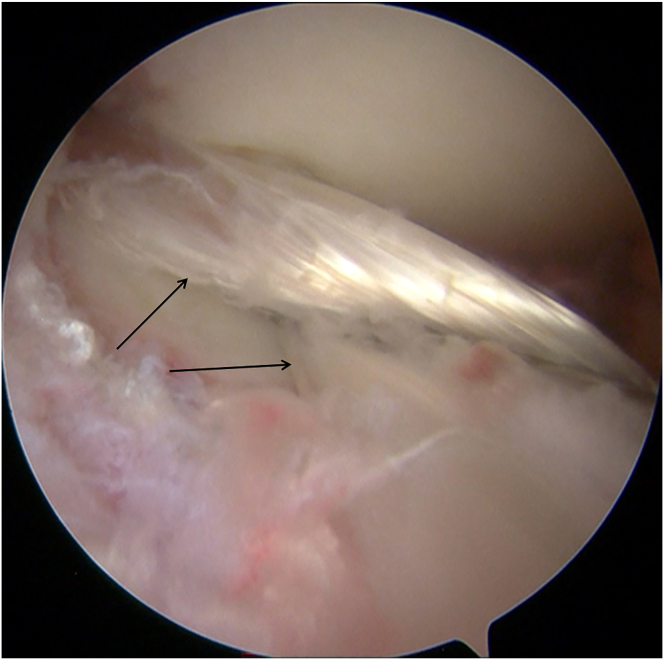
The graft is pulled into the joint (left knee).

The same procedure is applied to the anterior-root tibial tunnel. A second passing pin carrying a PDS-II loop is inserted into the joint, captured through the anterolateral portal, and used to shuttle the sutures from the graft’s anterior end through the anterior-root tunnel (Fig 6).

A probe confirms that the tendon graft courses circumferentially along the joint capsule. The high-strength sutures limbs from both ends of the graft exit their respective tibial tunnels and remain extracortical on the tibia, ready for final fixation.

### Suture Fixation

**Fig 7:**
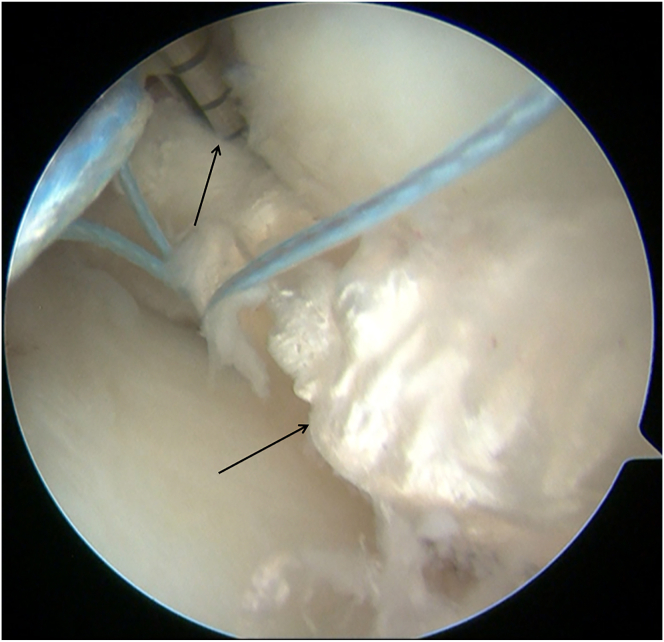
The all-inside meniscal repair device (20°; Rejoin) secures the graft to the posterior root remnant (left knee).

**Fig 8:**
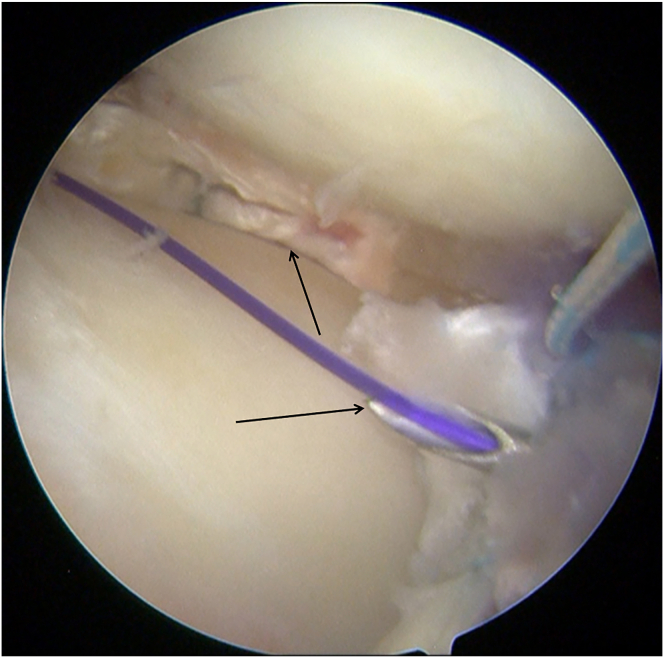
One outside-in stitch (Jinzhong) is placed at the anterior horn.

**Fig 9:**
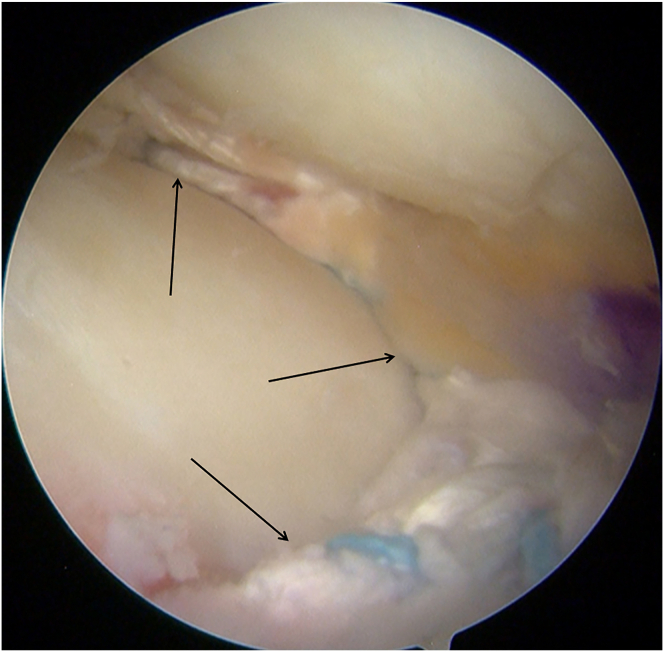
Final arthroscopic inspection (left knee, anterolateral portal) confirms that the tendon graft is firmly seated.

The all-inside meniscal repair device (20°; Rejoin) secures the graft to the posterior root remnant (Fig 7). Four additional all-inside sutures are placed sequentially from the posterior horn toward the meniscal body, anchoring the graft to the joint capsule along its posterior and midbody segments. One outside-in stitch (Jinzhong) is placed at the anterior horn to complete the circumferential construct, forming a stable, ring-shaped meniscal scaffold (Fig 8). Final arthroscopic inspection confirms that the tendon graft is firmly seated and assumes a meniscus-like contour (Fig 9). The extracortical high-strength limbs exiting the tibial tunnels are fixed to the tibial cortex with a PEEK suture anchor (4.75 mm; Rejoin) for supplemental fixation.

### Postoperative Assessment and Rehabilitation

The morphology and signal intensity of the APLT graft are evaluated by magnetic resonance imaging. The morphology and signal intensity of the APLT graft are similar to normal meniscus after surgery. The affected limb was fixed with a brace in an extended position for 4 weeks. The passive movement of the knee joint from 0° to 45° within 2 weeks after surgery. The passive range of motion should not exceed 90° within 3 to 4 weeks after surgery and is allowed full range activities after 4 weeks. The partial weight-bearing walking begins after 4 weeks, and complete load bearing starts at the sixth week after surgery.

## Discussion

Restoring circumferential (hoop) tension after subtotal or total meniscectomy is pivotal for re-establishing load sharing, protecting articular cartilage, and delaying osteoarthritic progression.^4^ Conventional options, including total meniscectomy, MAT, and synthetic scaffold implantation, have well-documented limitations.^5^ Meniscectomy rapidly relieves pain but increases tibiofemoral contact pressures and accelerates cartilage wear. Allograft transplantation is hampered by graft availability, cost, immune risk, and size-matching difficulties, whereas currently available scaffolds provide only partial restoration of biomechanics and require an intact meniscal rim.^6^

The APLT offers several advantages that directly address these shortcomings: (1) Adequate length and cross-section. The APLT averages 28 to 30 cm in length with a cross-sectional area comparable with that of the native lateral meniscus, allowing creation of a full-circumference graft without resorting to synthetic augmentation.^7^ (2) Superior tensile properties. Biomechanical testing shows that APLT autografts exhibit ultimate failure loads (≈600 N) that exceed those of semitendinosus and match fresh-frozen allograft tissue, conferring robust hoop-stress resistance.^8^ (3) Favorable biology. As an autologous graft, the APLT eliminates infectious and immunogenic risks inherent to allografts and promotes rapid revascularization and cellular repopulation, prerequisites for long-term survivability.^9^ (4) Minimal donor-site morbidity. Harvesting only the anterior (hemisection) of the APLT preserves ankle eversion strength; recent gait and isokinetic studies show no clinically meaningful loss of function after partial harvest.^10^

This dual-tunnel, circumferential reconstruction approach reproduces both anterior and posterior meniscal roots and secures the tendon along its capsular periphery with inside-out and outside-in sutures. This technique neutralizes meniscal extrusion—a common failure mode after root repairs—by converting axial loads into hoop stresses. Routing the graft in a smooth arc between the tunnels avoids the “killer-turn” effect that compromises graft longevity in transtibial pull-out repairs.

In contrast to MAT, our autograft is immediately available, size-matched, and inexpensive. Unlike scaffold procedures (e.g., Collagen Meniscal Implant [CMI; Stryker Corporation] or Actifit [Orteq Ltd.]), the peroneus longus tendon graft withstands compressive loading from the moment of implantation, thereby reducing the risk of early deformation or fragmentation. Early postoperative imaging in the present series confirmed anatomic restoration of meniscal volume without extrusion, and all patients achieved pain relief and functional improvement by 3 months. Although these results parallel or surpass MAT outcomes at similar time points, long-term follow-up will be required to determine chondroprotective efficacy.

Several caveats merit discussion. First, tendon tissue lacks the native fibrocartilaginous architecture of the meniscus; although remodeling toward a meniscus-like matrix has been shown in animal models, the extent of human conversion remains uncertain. Second, harvesting the APLT may not be appropriate in high-level athletes who depend heavily on ankle eversion strength. Third, because this was an early technical series without a control group, direct comparison with MAT or scaffold implants is not yet possible.

Circumferential lateral meniscal reconstruction using APLT provides an immediately available, mechanically robust, and biologically favorable solution for otherwise-irreparable lateral meniscal deficiency.^11^, ^12^, ^13^ The advantages and disadvantages of our technique are listed in Table 2. The technique effectively restores hoop tension, prevents extrusion, and offers a promising alternative to allograft transplantation and synthetic scaffolds. With careful patient selection and continued investigation, APLT autograft reconstruction may become a valuable addition to the armamentarium for joint-preserving knee surgery.

**Table 2:** Advantages and Disadvantages of the Technique

Advantages
Functional meniscal repair by restoring hoop tension and preventing extrusion
Autologous APLT graft eliminates immune response and ethical concerns
Favorable bone-tendon healing, promoting graft integration and long-term viability
Mechanical properties of APLT graft provide superior load distribution and resistance
Disadvantages
Requires a high level of surgical skill and precision for effective execution
Potential donor-site morbidity, especially for patients with high ankle demands
Limited long-term data regarding graft durability and long-term knee preservation
May not be suitable for athletes who rely heavily on ankle eversion strength
Requires careful patient selection and thorough preoperative evaluation

APLT, autologous peroneal longus tendon.

## Disclosures

The author (J.W.) declares that he has no known competing financial interests or personal relationships that could have appeared to influence the work reported in this paper.

## Supplementary Data

Video 1 In this Technical Note, the patient is in the supine position with the left knee flexed. Diagnostic arthroscopy is performed through the anterolateral portal and anteromedial portal. First, a standard anterolateral portal is established, and a 30° arthroscope (Smith & Nephew) is introduced into the knee. The diagnostic exploration of knee is implemented. The incarcerated portion is excised using a basket punch (Smith & Nephew). The remaining tear margins are debrided, the posterior root remnant preserved, and the highly vascular tibial attachment bed gently refreshed. The anterior half of the tendon is harvested (6-mm diameter tendon stripper; Smith & Nephew). The retrieved graft measures approximately 20 cm in length and 4 mm in diameter. A 3-cm longitudinal skin incision is made medial to the tibial tuberosity. Through the anteromedial arthroscopic portal, the tibial footprints of both the anterior and posterior roots of the lateral meniscus are identified. A tibial-tunnel ACL reconstruction guide (Rejoin) is introduced via the medial incision and positioned on the footprint of the posterior root and anterior root. After arthroscopic confirmation of the pin position, a 4.5-mm cannulated reamer (Rejoin) is used to create the posterior-root tibial tunnel. A passing pin (Smith & Nephew) loaded with a polydioxanone II (Ethicon) loop is advanced through the posterior-root tibial tunnel, leaving the suture loop inside the joint. Via the anterolateral working portal, a grasper retrieves the loop and delivers it outside the joint. A probe (Smith & Nephew) confirms that the tendon graft courses circumferentially along the joint capsule. Four all-inside sutures (20°; Rejoin) are placed sequentially from the posterior horn toward the meniscal body, anchoring the graft to the joint capsule along its posterior and midbody segments. One outside-in stitch (Jinzhong) is placed at the anterior horn to complete the circumferential construct, forming a stable, ring-shaped meniscal scaffold. The extracortical high-strength limbs exiting the tibial tunnels are fixed to the tibial cortex with a polyether ether ketone suture anchor (4.75 mm; Rejoin) for supplemental fixation.
